# Irruption of Network Analysis to Explain Dietary, Psychological and Nutritional Patterns and Metabolic Health Status in Metabolically Healthy and Unhealthy Overweight and Obese University Students: Ecuadorian Case

**DOI:** 10.3390/nu16172924

**Published:** 2024-09-01

**Authors:** María Alejandra Aguirre-Quezada, María Pilar Aranda-Ramírez

**Affiliations:** 1 Nursing Career, Catholic University of Cuenca, Azogues 010107, Ecuador; maaguirreq@ucacue.edu.ec; 2 Faculty of Pharmacy, University of Granada, 18071 Granada, Spain

**Keywords:** Gaussian graphic model, exploratory analysis, dietary nutritional patterns, metabolically healthy obesity, metabolically unhealthy obesity

## Abstract

Background. The association between dietary nutritional patterns, psychological factors, and metabolic health status has not been investigated in university students. There are studies that include numerous variables to test hypotheses from various theoretical bases, but due to their complexity, they have not been studied in combination. The scientific community recognizes the use of Gaussian graphical models (GGM) as a set of novel methods capable of addressing this. Objective. To apply GGMs to derive specific networks for groups of healthy and unhealthy obese individuals that represent nutritional, psychological, and metabolic patterns in an Ecuadorian population. Methodology. This was a quantitative, non-experimental, cross-sectional, correlational study conducted on a sample of 230 obese/overweight university students, selected through a multi-stage random sampling method. To assess usual dietary intake, a Food Frequency Questionnaire (FFQ) was used; to evaluate psychological profiles (anxiety, depression, and stress), the DASS-21 scale was employed; blood pressure and anthropometric data were collected; and insulin levels, lipid profiles, and glucose levels were determined using fasting blood samples. The International Diabetes Federation (IDF) criteria were applied to identify metabolically healthy and unhealthy individuals. Statistical analysis relied on univariate methods (frequencies, measures of central tendency, and dispersion), and the relationships were analyzed through networks. The Mann-Whitney U test was used to analyze differences between groups. Results. In metabolically unhealthy obese individuals, GGMs identified a primary network consisting of the influence of waist circumference on blood pressure and insulin levels. In the healthy obese group, a different network was identified, incorporating stress and anxiety variables that influenced blood pressure, anthropometry, and insulin levels. Other identified networks show the dynamics of obesity and the effect of waist circumference on triglycerides, anxiety, and riboflavin intake. Conclusions. GGMs are an exploratory method that can be used to construct networks that illustrate the behavior of obesity in the studied population. In the future, the identified networks could form the basis for updating obesity management protocols in Primary Care Units and supporting clinical interventions in Ecuador.

## 1. Introduction

Obesity is a highly prevalent condition that affects global public health, increasing the need for economic resources to cover treatment costs [1]. One of the main concerns is that the overweight population has continued to rise, with no country experiencing a reduction, and the increase is more pronounced in developing nations. For a long time, international organizations have been warning society about the obesity crisis and its numerous effects on health. After the COVID-19 pandemic, it was shown that those affected by obesity had a higher probability of death [2].

Over the past 45 years, global obesity has tripled; it is estimated that 39% of people over 18 years old are overweight and 13% are obese [1]. It has been determined that obesity is more prevalent in men than in women. Accordingly, the World Obesity Federation report warns that without real public prevention policies, more than half of the global population will suffer from overnutrition by 2025 [3].

Preventing obesity is not only a public health interest but also a means to reduce the burden on already overwhelmed healthcare systems. Epidemiological data emphasize its effects; the medical expenses for an obese person are 36% higher than those for a person of healthy weight, thus creating an economic problem that demands additional budgetary costs [4].

Accumulated evidence has shown that overnutrition is a well-established risk factor for a wide variety of complications, such as prediabetes, type 2 diabetes, dyslipidemia, hypertension, cardiovascular diseases, non-alcoholic fatty liver disease, and osteoarthritis [5,6]. Each year, 15 million people between the ages of 30 and 69 die from non-communicable diseases (NCDs) worldwide; more than 85% of these “premature” deaths occur in low- and middle-income countries. Consequently, it is necessary to implement healthcare interventions to prevent and treat overweight and obesity, as well as their associated complications [7].

In Latin America and the Caribbean, nearly 2.8 million people died from non-communicable diseases (NCDs). Therefore, the World Health Organization (WHO) recommends maintaining a healthy diet throughout the life cycle to prevent them [8]. However, these complications do not develop in the entire obese population, and some may be free from these cardiometabolic factors. Hence, obese or overweight individuals without risk factors such as hypertension, dyslipidemia, insulin resistance, or prediabetes are referred to as metabolically healthy obese (MHO), while those who present the mentioned risk factors are referred to as metabolically unhealthy obese (MUO) [9].

Recognizing obesity as a multifactorial condition, there is a cause with limited evidence, and the reality in Ecuador is no different. The Ministry of Public Health has reported that the prevalence of overweight and obesity in the adult population exceeds 50% [10], despite the implementation of national prevention programs, and the prevalence among university students varies around 30%. In the country, there are over 600,000 university students [11], and the results of previous studies show that the dominant risk factors for obesity are diet and low levels of physical activity. The relevance of the proposed research seeks to demonstrate the link between nutritional, psychological, and metabolic variables, which has been little studied internationally and has not been studied at all nationally (in Ecuador).

The study aims to apply GGMs to derive specific networks for groups of healthy and unhealthy obese individuals that represent the nutritional, psychological, and metabolic patterns in an Ecuadorian population.

The findings reveal that more than 1.9 billion students worldwide are overweight, and over 650 million are obese, alarming figures regarding the impact on the quality of life of the young population [12]. The diet of university students is characterized by being monotonous, of low nutritional quality, and often does not meet daily nutritional requirements. Similarly, university life presents a set of situations considered challenging (changes in schedule, exams, moving to another place, peer groups, etc.), often associated with psychological problems that contribute to risk characteristics affecting diet [13].

On the other hand, it should be considered that evidence shows that obesity and depression have been studied frequently, although it is not yet fully understood how they are linked. Some research suggests that depression is more prevalent among obese people, particularly women [14,15].

However, previous studies have had limitations in their analysis. Therefore, there is a need for a new method that allows researchers to “intuit” patterns in their data sets. Exploratory analyses are useful for examining similarities and differences in relationships between variables across multiple groups [16].

It is evident, then, that the use of Gaussian graphical models provides a comprehensive, easy-to-understand overview of the relationships between variables included in a study [17]. Based on this premise, this study presents the application of Gaussian graphical models in a study of obesity and overweight among university students in Ecuador. Furthermore, these graphical models can reveal relationships between variables that published studies have not described or theorized, such as relationships derived from different theories that had not been previously examined in combination, or they may reveal others that researchers had not anticipated or theorized.

## 2. Materials and Methods

### 2.1. Design and Participants

Epidemiological, non-experimental, cross-sectional, cross-sectional, correlational, quantitative, network analysis study, developed in the city of Azogues, Ecuador. The unit of analysis was the overweight and obese students of the Catholic University of Cuenca, which corresponds to 230 students, estimated based on a prevalence of 30% of MUO among overweight and obese Ecuadorian university students of different socioeconomic levels [18], power of 80%, desired confidence interval of 0.95, type I error of 0.05 and precision (d) of 7%. According to the university age range [19], participants between 18 and 29 years of age, were chosen through a multistage mass sampling approach. 

### 2.2. Inclusion and Exclusion Criteria

All students, who voluntarily agreed to sign the informed consent form, were included, excluding students with pregnancy, with endocrine or genetic disorders (hypothyroidism, type 1 diabetes mellitus, and Cushing’s syndrome), following a weight-loss diet, or those taking mineral and vitamin supplements, or medications that could affect blood glucose, lipid profile, body weight or blood pressure.

### 2.3. Data Acquisition

Information was collected using observation and survey techniques.

#### 2.3.1. Anthropometry

The dependent variable was the nutritional status of the students, obtained through the body mass index (BMI), which is an indicator of the relationship between weight in kg and height in m^2^, used to classify overweight and obesity, according to the WHO [20]: Normal weight = BMI less than 25 kg/m^2^. Overweight = BMI equal to or greater than 25 kg/m^2^. Obesity = BMI equal to or greater than 30 kg/m^2^.

#### 2.3.2. Socioeconomic Variables

The independent variables were categorized as socioeconomic, the Gaffar scale was applied to evaluate the socioeconomic status, for the sociodemographic variables, age, sex at birth, marital status, academic unit, career, and study schedule of the participants were collected through a questionnaire. 

#### 2.3.3. Assessment of Dietary Intake

The dietary intake of the participants was measured through a food frequency questionnaire (CFCA) [21]. A trained nutritionist completed the questionnaires and asked people to indicate the frequency of food consumption (daily, weekly, or monthly) and the amount consumed (based on common standard portion sizes) in the last twelve months. Then, using home measurements, all reported values were converted into grams per day. Subsequently, the total energy and nutrient intake of each individual was calculated by summing the energy and nutrients from all foods. To derive nutrient intake, grams of food consumption were entered into Nutrimind software 2024 [22].

#### 2.3.4. Cardiometabolic Risk Factors

The evaluation of anthropometric indicators and cardiometabolic risk factors was carried out by a previously trained team from the nursing faculty, who recorded the anthropometric indices of all the participants. Weight was measured with a calibrated electronic scale (BCS-G6) (closest to 0.1 kg). Standing height was recorded with a stadiometer (nearest 0.1 cm). Waist circumference (WC) was measured twice for each participant and the mean value of two assessments was considered WC [23]. After a 5-min rest, diastolic blood pressure (DBP) and systolic blood pressure (SBP) were recorded twice with a 15-min recovery interval, on the right arm [24].

The average of two measurements was considered in the analysis. To determine the biochemical values, venous blood samples were obtained in a seated position, after a twelve-hour fast. Fasting blood glucose concentration, lipid profile, and insulin concentrations were determined. To estimate insulin resistance, Homeostasis Model Assessment Insulin Resistance (HOMA-IR) was calculated using the following formula: HOMA-IR = [insulin (µIU/mL) × glucose (mg/dL)]/405 [25].

In the evaluation of metabolic status, two methods were applied to classify participants into MUO and MHO [9]. The first method was based on the modified International Diabetes Federation (IDF) criteria, whereby students with two or more of the following CRFs were considered as MUO: (1) increased triglycerides (≥150 mg/dL), (2) elevated fasting blood glucose (≥126 mg/dL), (3) decreased HDL-c (<45 mg/dL), and (4) elevated blood pressure (≥120/80 mmHg) [26]. In this method, those with one or no risk factors will be considered college MHO.

In the second classification, insulin resistance, defined by the HOMA-IR score, was added to the IDF criteria, which was applied in the first classification. Thus, students with HOMA-IR score ≥ 3 and two or more metabolic risk factors were considered MUO individuals, and those with HOMA-IR < 3 were considered MHO. 

The cutoff value of 3 [27,28] for HOMA-IR was selected based on several previous studies among college students.

#### 2.3.5. Psychological Pattern

For the evaluation of psychological variables, the abbreviated version of the Depression Anxiety and Stress Scales (DASS-21) was used [29]. The three-dimensional self-report scales assessed the presence and intensity of affective states of depression, anxiety, and stress. Each item was answered according to the presence and intensity of each symptom on a Likert-type response scale from 0 to 3 points. Each scale has seven items, and its total score is calculated with the sum of the items belonging to that scale and ranges from 0 to 21 points.

#### 2.3.6. Level of Physical Activity

Finally, the level of physical activity of each participant was assessed by the Physical Activity Questionnaire (IPAQ), which included nine questions on various activities during weekdays and weekends. Based on total scores, undergraduates were classified as active (score ≥ 3), not very active (3 < score ≤ 2), or sedentary (or no regular weekly activity) (score < 2) [30].

### 2.4. Data Analysis

The data collected were tabulated and analyzed in SPSS^®^, v27.0 and Jamovi v2.2.2 software [31]. The Kolmogorov-Smirnov normality test was applied, showing a nonparametric distribution for the variables because they did not meet the normality assumptions. 

Was used to apply the Gaussian graphical model on the dataset. We computed mean scores for items assumed to be belonging to the same scale to form variables. To estimate the Gaussian graphical model, we first need to estimate the correlation matrices at the item and scale level, respectively.

Statistical analysis relied on univariate methods (frequencies, measures of central tendency, and dispersion), and the relationships were analyzed through networks. The Mann-Whitney U test was used to analyze differences between groups.

Then, using the estimated correlation matrices as input, the Gaussian graphic model was estimated using the Glasso algorithm [30]. The graphs were then visualized with the Jamovi v2.2.2 matrix package. In the Gaussian graphic, variables that are strongly correlated are placed spatially close to each other using the Fruchterman-Reingold algorithm of Fruchterman Reingold, which does not imply that they are semantically or conceptually similar.

The strength of the graphs is their ease of interpretation. The thickness of the line indicates the strength of the relationship. Green lines indicate positive partial correlation coefficients and red lines indicate negative partial correlations. In areas, where partial correlations with an absolute value less than 0.1 are not displayed.

The correlation matrix is attached in the Supplementary Materials section.

This study has the authorization of the Committee on Ethics and Research in Human Subjects (CEISH), Catholic University of Cuenca, with code CEISH-045, approval date: 27 July 2023.

## 3. Results

The information in Table 1, Table 1 shows the descriptive statistics, and the analysis confirms a non-parametric distribution, as the assumptions of normality are not met; thus, the statistics used are adjusted to these conditions.

Ecuadorian students with obesity present several risk indicators for metabolic and cardiovascular diseases compared to a healthy population. This underscores the need to reconsider the current interventions in the country by the health authorities.

When analyzing the intake pattern, it is shown that the consumption of vitamins is lower for Vitamin D, Vitamin E, Pantothenic Acid, Biotin, and Folic Acid. Regarding mineral intake, consumption of Magnesium, Iron, and Zinc is determined to be below the recommended levels. Of particular concern is the excessively high sodium intake observed among these students with overweight and obesity issues.

In terms of psychological patterns, the average scores on the depression, stress, and anxiety tests place the study population in the moderate to severe categories, highlighting a concerning mental health situation.

There is information comparing the MHO and MUO groups in Table 2. The results show significant differences in several variables indicated a higher metabolic and cardiovascular risk in the MUO group. The findings highlight the metabolically unhealthy obese have higher weight, are smaller, have higher BMI and their waist circumference on average is higher which is associated with higher cardiovascular and metabolic risk, higher diastolic and systolic blood pressure, higher fasting glucose, insulin, cholesterol, triglycerides, HOMA, higher dietary cholesterol, protein, Thiamine, Niacin, B5, B6, Sodium, Iodine, Copper, Manganese. 

As opposed to showing a lower HDL value, a lower intake of kilocalories, fiber, fat, water, Vitamin A, Cobalamin, Vitamin D, Folic Acid, Potassium and Calcium. None of the participants in the MHO group had high systolic blood pressure values. 

There is a greater conflict with psychological patterns in the MUO population. The differences identified between the groups, generates a bibliographic base in the young Ecuadorian population. 

**Figure 1:**
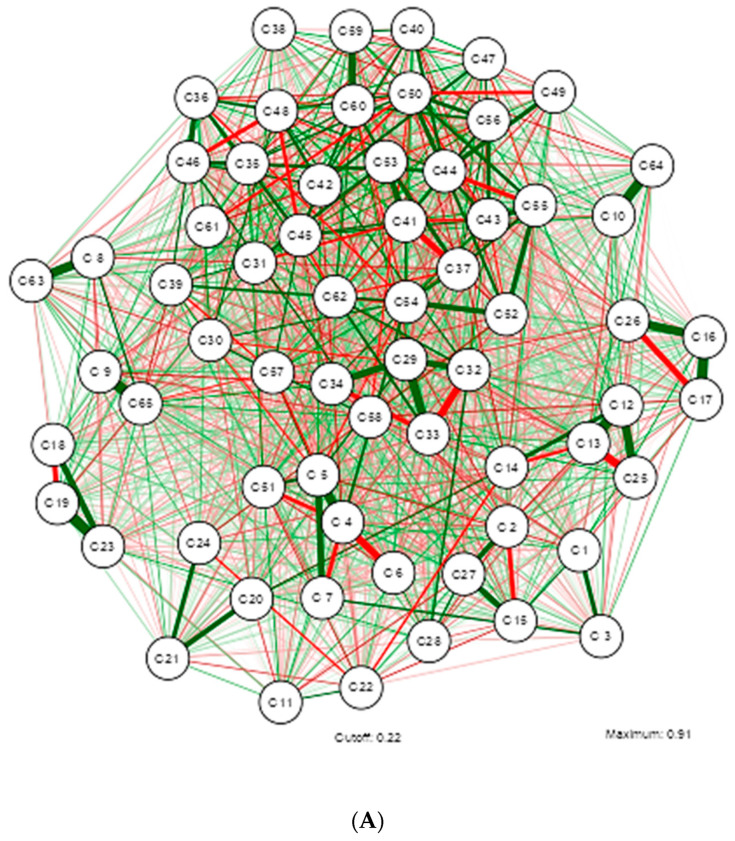

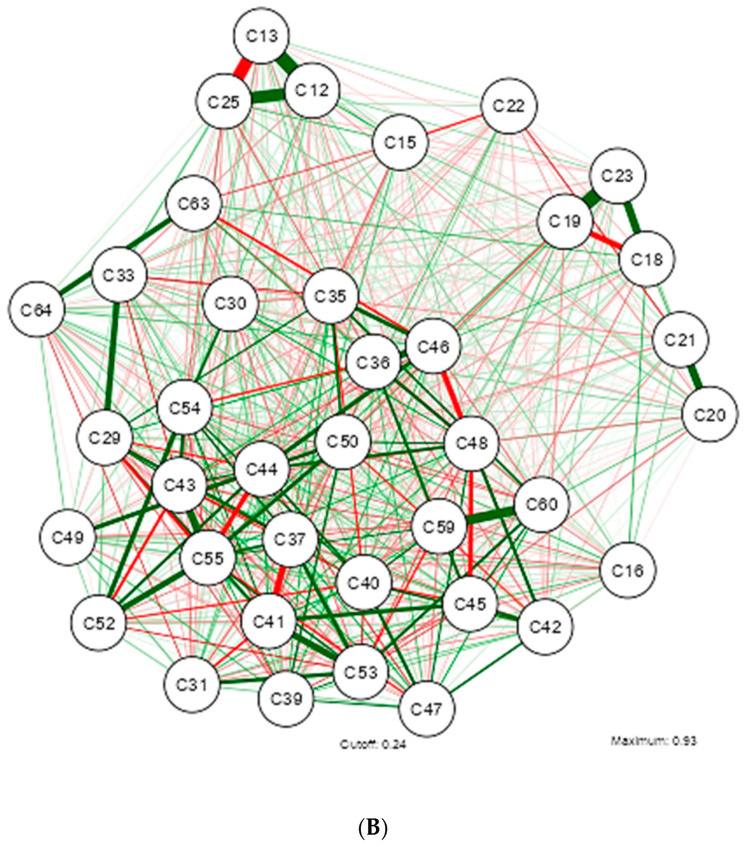

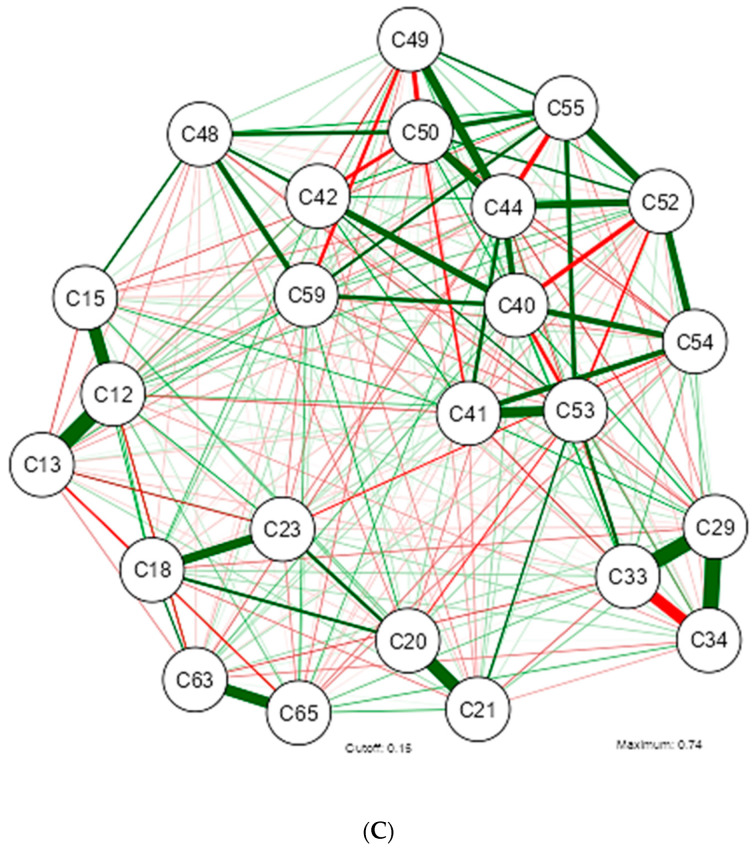

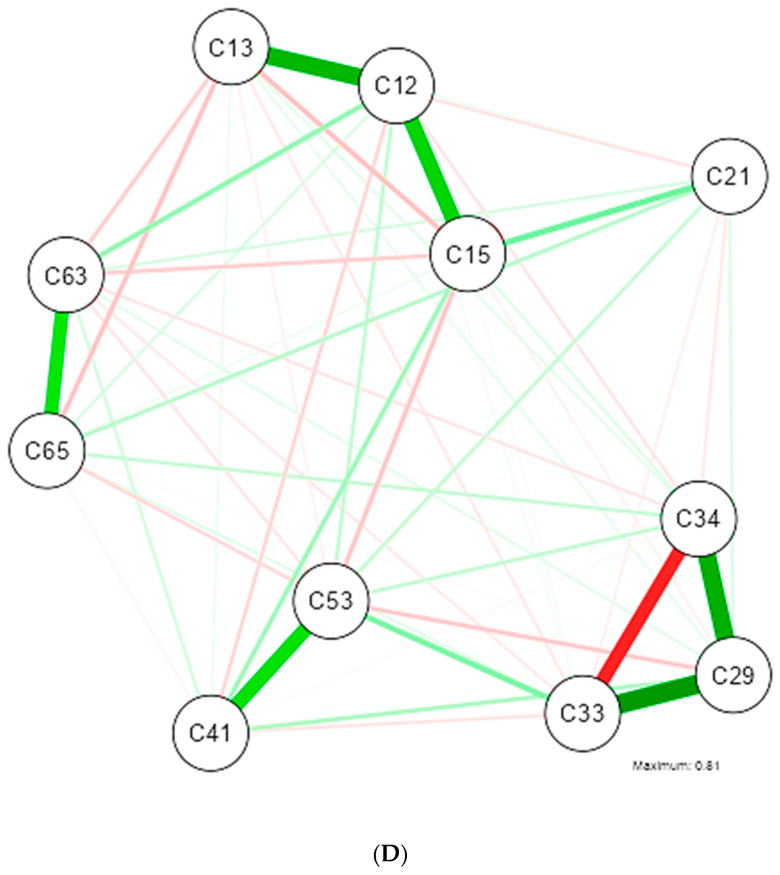
Illustrating the estimation of a Gaussian graphical model using the extended Bayesian information criteria (EBIC) and the glasso algorithm. Note that the EBIC optimally sets the turning parameters such that strong relationships are retained in the graph and weak relationships are set to zero. Note that the positive relationship is marked by the green colored lines and the inverse by the red colored lines; the thicker the line the stronger the relationship of variables. (**A**) Gaussian graphical model with analysis of all Dietary, Psychological, and Metabolic Health Status Nutritional Patterns in Overweight and Obese University Students. (**B**) Gaussian graphical model after applying correlation Rho (0.400–0.599) in study variables. (**C**) Gaussian graphical model after applying correlation Rho (0.600–0.799) in study variables. (**D**) Gaussian graphical model after applying correlation Rho (0.800–0.999) in study variables.

The GGM analysis identified one main network and three smaller networks formed by strongly correlated variables, as described in Figure 1. The meaning of each code assigned to the study variables can be consulted in the Appendix A described in Table A1.

The main network provides an initial insight into the phenomenon under study and one-dimensionality, which aims to test whether the nodes presumed to measure the same variable are indeed related by analyzing 65 variables. Nine sub-networks were identified, grouped around dietary patterns.

The network revealed that an older age of the participants increases waist circumference (WC), which in turn correlates with higher scores of stresses, anxiety, and depression, but this relationship is inverse with sex at birth. The findings in the dietary patterns indicate that higher daily carbohydrate intake is associated with lower protein intake. A positive association is noted between Vitamin D, Calcium, and Phosphorus, while a negative correlation is identified between Vitamin C and Magnesium.

The network model suggests that variables related to blood pressure and psychological patterns are strongly interconnected with variables of body composition, metabolic parameters, and nutrient intake. This analysis can help identify potential areas for intervention to improve both physical and mental health within the health protocols for managing obesity in Primary Care Units in Ecuador, as shown in Network A.

Network B, which explores the moderate relationship described by a Rho of 0.400–0.599, includes 43 variables. The identified findings contribute to a better understanding of the disease within the Ecuadorian population and could aid in the education of undergraduate and graduate students in health-related fields.

The grouping dynamics around dietary patterns are maintained, but with the presence of four sub-networks. In the formation of the sub-networks, it is observed that higher WC increases blood cholesterol levels, but decreases HDL levels. Higher glucose levels are associated with higher scores on the depression test. The network shows a positive relationship between Vitamin C and Folic Acid, and between Cobalamin and Calcium.

The variables with a high correlation that explain overweight and obesity in Ecuadorian university students include 24 variables and 5 sub-networks, as explained in Network C. It is important to note that this network no longer includes stress test scores, HDL, insulin, dietary cholesterol, as well as several nutrients.

The study population findings reveal that higher glucose levels are associated with higher depression test scores among students. Additionally, high Magnesium intake is negatively correlated with Vitamin C intake. Students with larger waist circumferences present higher blood cholesterol and triglyceride levels. Similarly, higher anxiety test scores are associated with elevated blood cholesterol levels, and increased Thiamine intake is related to lower Calcium intake.

Finally, Network D describes the variables that maintain a very high statistical relationship, consisting of 11 variables that could predict overweight and obesity in the Ecuadorian university population.

The findings, which contribute to the literature in Ecuador, show that higher fat intake increases WC, which in turn increases triglyceride levels. Students with higher body weight score higher on the depression test, but those with greater height score lower on the anxiety test. In this population, higher daily calorie intake is also associated with increased carbohydrate and fat consumption. The strong connections between weight, height, WC, fat intake, depression, and anxiety suggest that these factors are strongly interrelated and could correspond to primary factors influencing obesity.

However, the study aims to generate a contribution so that researchers can have information intake, metabolic health, physical activity, psychological and sociodemographic variables behave in the 2 groups of MUO and MHO. 

**Figure 2:**
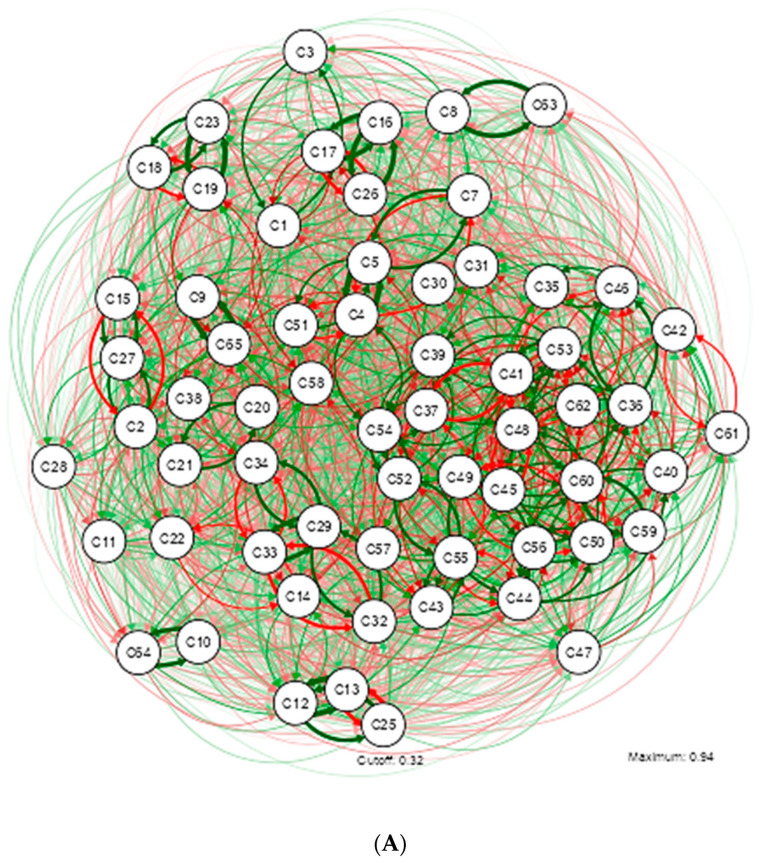

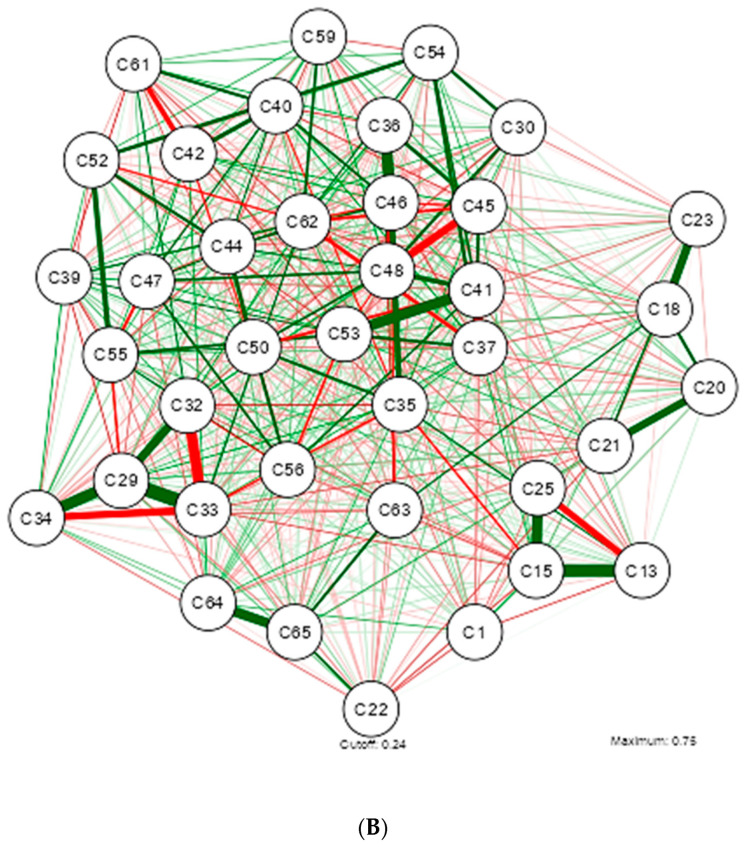

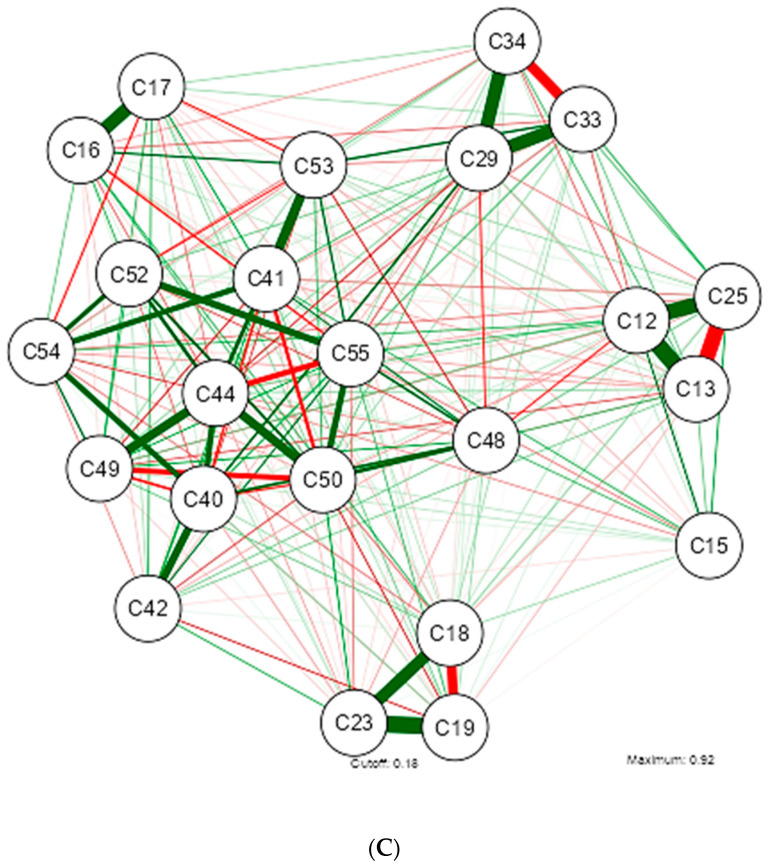

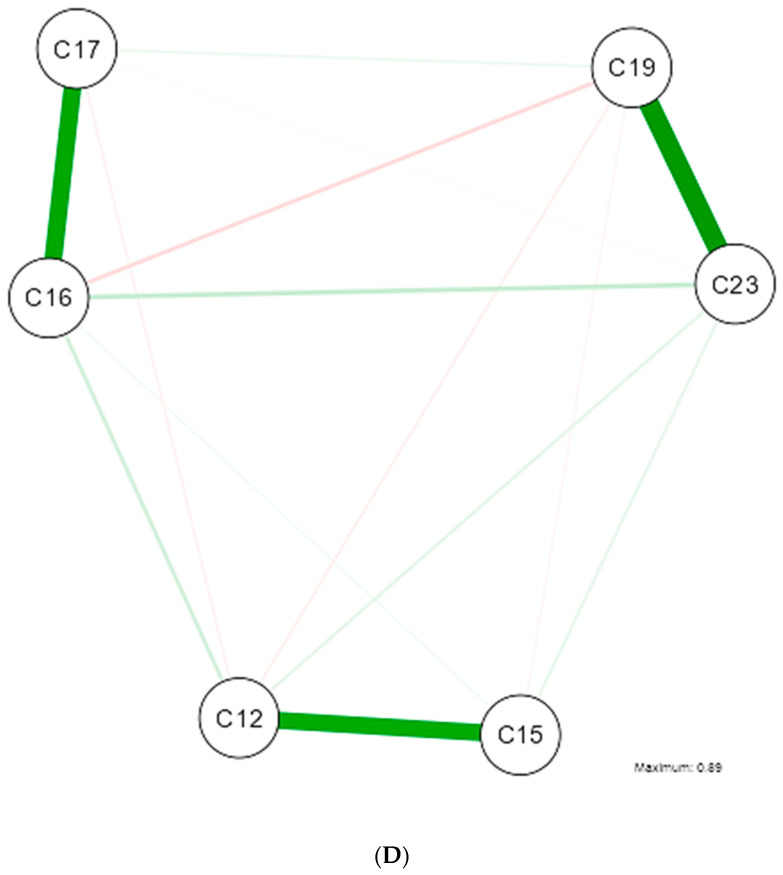
Illustrating the estimation of a Gaussian graphical model using the extended Bayesian information criteria (EBIC) and the glasso algorithm. Note that the EBIC optimally sets the turning parameters such that strong relationships are retained in the graph and weak relationships are set to zero. Note that the positive relationship is marked by the green colored lines and the inverse by the red colored lines; the thicker the line the stronger the relationship of variables. (**A**) Gaussian graphical model with analysis of all Dietary, Psychological, and Metabolic Health Status Nutritional Patterns in MUO. (**B**) Gaussian graphical model after applying Rho correlation (0.400–0.599) in MUO study variables. (**C**) Gaussian graphical model after applying Rho (0.600–0.799) in MUO study variables. (**D**) Gaussian graphical model after applying correlation Rho (0.800–0.999) in MUO study variables.

The relationship of the variables for the MUO group is described in Figure 2.

The study of metabolically unhealthy obesity is of particular interest to the nutritional epidemiology of Ecuador. The main network highlights a different grouping dynamic compared to what was identified in Network A from Figure 1. The relationship between these variables is not linear but rather presents complex interactions, resulting in a grouping of 7 interrelated sub-networks, as described in Network A from Figure 2.

The network reveals that in the MUO group, as age increases, so does blood pressure. Additionally, there is a positive relationship between WC, glucose, and levels of stress, anxiety, and depression. These results suggest that university students with obesity may have poorer mental health.

A predictable result was the positive relationship between sodium intake and blood pressure, emphasizing the importance of diet in its regulation, especially considering the evidence that greater WC, regardless of total weight, is a strong predictor of hypertension. Regarding the consumption of macronutrients and micronutrients, the intake of fiber, proteins, carbohydrates, and fats also shows positive relationships with BMI.

Network B, shows a complex interrelation among multiple variables, indicating the multifactorial nature of obesity. The moderate relationship described by a Rho of 0.400–0.599 identifies 5 sub-networks and 35 variables. Subjects with higher BMI values tend to have a larger WC. These findings suggest that these variables could be key factors significantly influencing the MUO condition in Ecuador.

Higher levels of polyunsaturated fatty acids in the diet are associated with higher levels of insulin resistance. This finding could have implications for better understanding the dietary and metabolic patterns in this group of patients. It would be important to further explore this relationship and investigate the possible underlying mechanisms that explain how polyunsaturated fatty acids are associated with insulin resistance.

Regarding micronutrients, some less-studied relationships are highlighted: higher calcium intake is positively associated with riboflavin, but there is an inverse relationship between (Vitamin D—Pantothenic Acid) and (Pyridoxine—Manganese). This information could be used to develop predictive models that help anticipate the risk and progression of metabolically unhealthy obesity in specific populations.

Network C, describes the variables with a strong relationship in students categorized as MUO. The new dynamic includes 5 sub-networks, which are grouped around micronutrients. In this study population, higher glucose levels are positively associated with diastolic blood pressure and weight. The consumption pattern shows that higher fat intake leads to a larger WC. Higher insulin levels show an inverse relationship with pyridoxine. Additionally, when Vitamin C intake is higher, magnesium intake tends to be lower, or higher riboflavin intake leads to higher calcium intake.

Network D, significantly contributes to the understanding of MUO obesity in Ecuador. These correlations indicate that blood pressure variables, insulin resistance indicators, and anthropometric measures are strongly related in patients in the MUO group. This finding is vital for Ecuadorian literature and highlights the importance of monitoring these parameters in the evaluation and management of MUO patients for comprehensive care.

**Figure 3:**
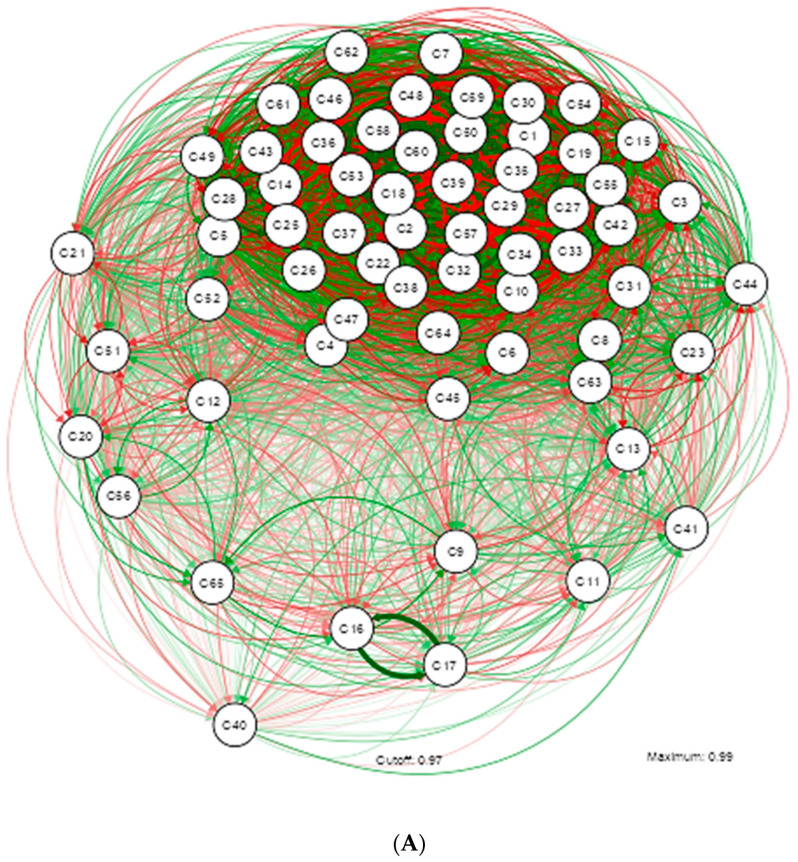

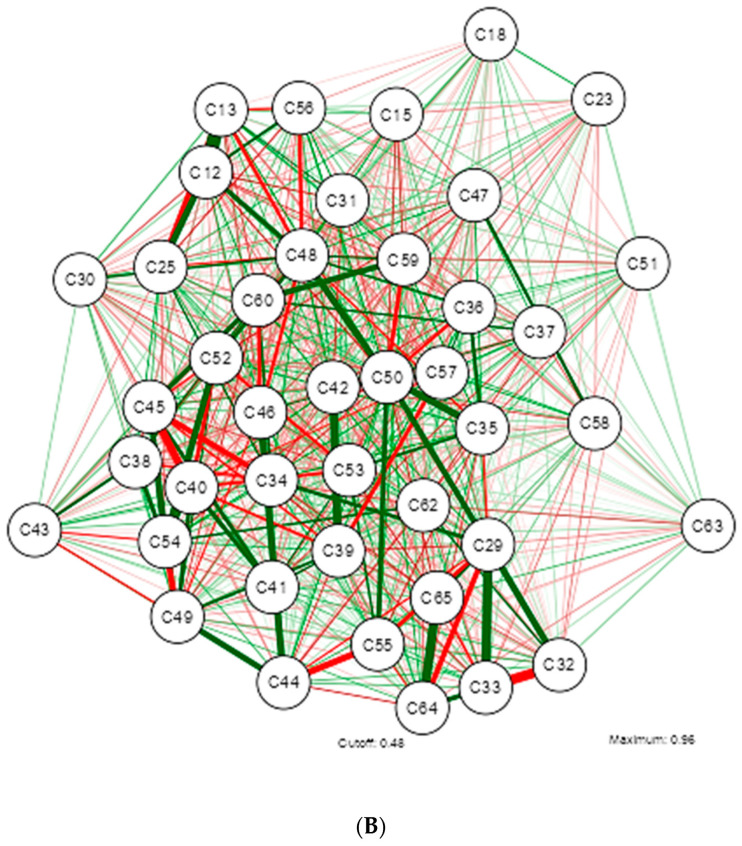

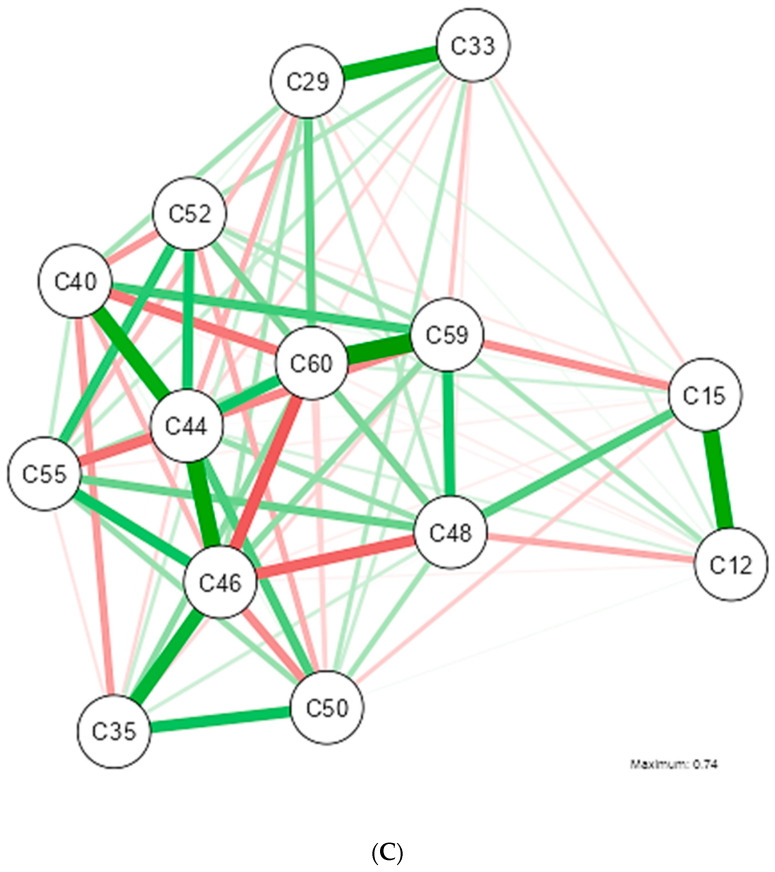

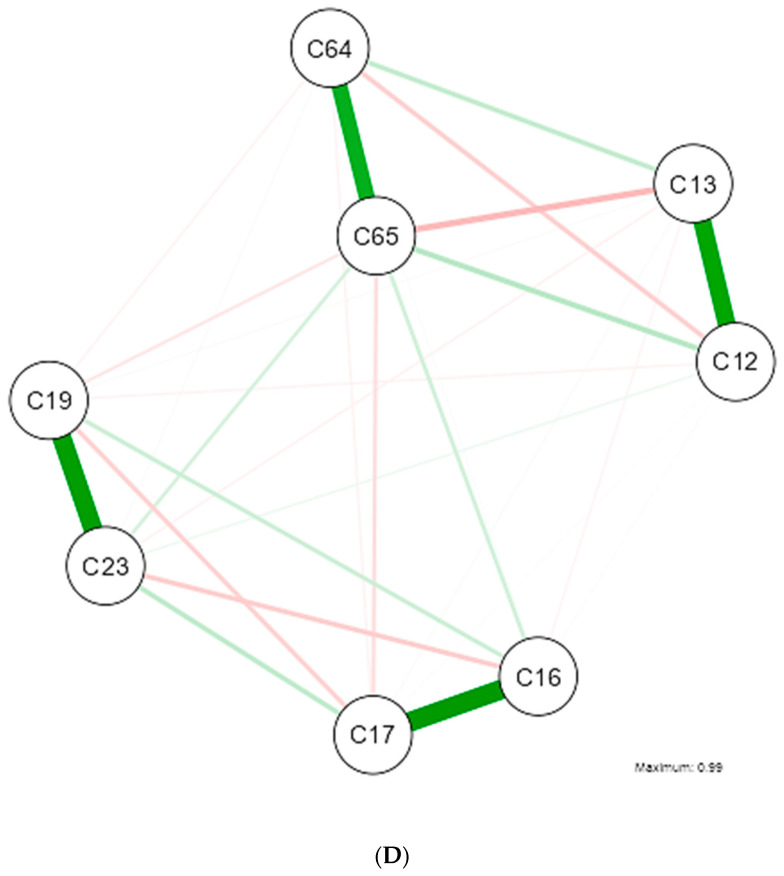
Illustrating the estimation of a Gaussian graphical model using the extended Bayesian information criteria (EBIC) and the glasso algorithm. Note that the EBIC optimally sets the turning parameters such that strong relationships are retained in the graph and weak relationships are set to zero. Note that the positive relationship is marked by the green colored lines and the inverse by the red colored lines; the thicker the line the stronger the relationship of variables. (**A**) Gaussian graphical model with analysis of all Dietary, Psychological, and Metabolic Health Status Nutritional Patterns in MHO. (**B**) Gaussian graphical model after applying Rho correlation (0.400–0.599) in MHO study variables. (**C**) Gaussian graphical model after applying Rho (0.600–0.799) in MHO study variables. (**D**) Gaussian graphical model after applying correlation Rho (0.800–0.999) in MHO study variables.

The main network of this group of studied subjects reflects multiple factors that contribute to maintaining a metabolically healthy state despite obesity. This analysis facilitates the understanding of other dimensions, allowing for a more holistic and effective approach to the intervention and prevention of this condition, described in Figure 3.

The grouping of sociodemographic, dietary, and metabolic variables is marked by a pronounced subnetwork; however, blood pressure, riboflavin, depression, stress, sodium, iron, physical activity level, and cholesterol deviate from this trend.

The psychological pattern shows a significant connection with various metabolic and anthropometric variables, but the network does not suggest a predominant negative influence on insulin and blood pressure. This indicates that, although these individuals experience stress and anxiety, their negative effects may be mitigated by other factors or healthy behaviors. A dense interconnection between vitamins and minerals is observed, suggesting that a diet rich in micronutrients could play a crucial role in protecting against metabolic complications associated with obesity.

The density and complexity of the connections in the network reflect how challenging it is to study MHO. The interaction of multiple variables suggests that it is not a single factor protecting these individuals, but rather a combination of behaviors, genetic factors, and dietary habits that contribute to maintaining a healthy profile. The numerous positive connections and the absence of strong negative correlations suggest that these individuals are protected by a combination of factors that keep their metabolic health in balance, as described in Network A.

The variables with a moderate relationship include factors that have an indirect or intermediate influence on obesity. Several inverse relationships are highlighted, which have not been determined in the other networks. This suggests that there is a considerable but not decisive influence on metabolically healthy obesity. For example, psychological patterns or metabolic health indicators may not directly cause obesity but contribute significantly when considered alongside other factors, as shown in Network B.

However, despite not yet developing cardiovascular complications, it is observed that students with high levels of stress also tend to experience high levels of anxiety, and higher glucose levels are associated with larger WC.

A complex network of interactions between various vitamins and minerals is observed. The findings show inverse relationships between the consumption of (kilocalories—stress), (cobalamin—biotin), and (vitamin C—magnesium). Sodium and potassium levels show important relationships, possibly reflecting their role in blood pressure and cardiovascular function. The presence of correlations between folic acid and other markers suggests its relevance to the overall health of these students.

The variables with a strong relationship, described in Network C, have a direct and significant impact on metabolically healthy obesity. The analysis reveals underlying mechanisms crucial for understanding the condition. A high correlation between BMI and triglyceride levels indicates a metabolic predisposition to obesity, which may be modulated by specific interventions in diet and exercise. There is a strong positive connection between calorie intake and grams of carbohydrates per day. This nutrient represents an important source of calories in the diet, but it is a modifiable factor, and its effect could reverse the identified relationship between higher body weight and increased WC.

To understand the influence of vitamins and minerals on the metabolic state of these individuals, vitamin E, an antioxidant, shows several positive connections with vitamin C and magnesium. Potassium is important for regulating blood pressure and fluid balance in the body, and its relationship with other variables reflects the need to maintain an adequate electrolyte balance to support cardiovascular health, even in obese individuals. Thiamine shows a relationship with other variables, suggesting an impact on metabolic efficiency. Its relationship in the network may reflect its role in overall health and metabolism.

The variables with very high relationships are critical determinants of metabolically healthy obesity. Their analysis allows for the identification of the most influential factors and the development of precise intervention strategies. It is important to highlight the predictive power of the variables included in the network. High levels of insulin and glucose suggest insulin resistance, which is a major factor in metabolic obesity. Identifying these relationships allows for the design of specific intervention programs that directly address these critical factors, as identified in Network D.

In a population with metabolically healthy obesity, it is observed that the psychological pattern includes stress and anxiety, but not depression, identifying a difference with the MUO group. The thickness of the green line between the insulin/HOMA variables and blood pressure suggests that there is no strong correlation. This would represent a positive signal, indicating that despite obesity, these students do not show signs of hypertension induced by insulin resistance.

Although no direct relationship is observed between stress and anxiety with metabolic markers such as insulin or blood pressure, these factors could be areas of concern that require further research. Stress is positively related to anxiety and height, but inversely related to weight. Insulin shows a positive relationship with diastolic blood pressure but an inverse relationship with systolic pressure.

Overall, despite obesity, the students appear to maintain a relatively healthy metabolic profile with typical correlations among the measured variables. However, high levels of anxiety and stress are very complex factors.

## 4. Discussion

This work investigated GGMs, an additional exploratory method for analyzing dietary patterns, psychological aspects, and metabolic health in university students classified as MUO and MHO. This approach, already used in oncology [32], nutritional dietetics [33], imaging [34], psychology [17], pharmacology [35], omics [36] and genetics [37], is presented as an innovative tool for studying these phenomena, allowing the identification of latent structures in the study variables related to overweight and obesity through the construction of networks. This method also reduces subjectivity in data analysis and reveals easily interpretable internal structures within the anthropometric, psychological, metabolic, and dietary data, which are visualized as networks related to obesity behavior [38].

GGMs demonstrate their ability to distinguish between direct and indirect associations among the selected categories of variables, based on the correlation matrix and the strength of the relationships. The networks obtained in this study are consistent with the variable relationships observed in other studies [39,40,41], suggesting that they adequately reflect the impact of obesity on the health patterns of the young population studied.

Various future approaches could continue to explore the identified networks. Conditional independence could be used to predict probabilities of consumption or mental illness diagnoses in individuals, which would be useful for designing comprehensive management protocols that adjust the probabilities of certain behaviors, thereby contributing to assessing the impact of healthy practices promoted by health authorities.

Additionally, the networks reveal a strong positive association in the MUO group between waist circumference (WC), blood pressure, and insulin. In contrast, in the MHO group, stress and anxiety variables influence blood pressure, anthropometry, and insulin—a finding not previously described in Ecuadorian literature. This is significant, as promoting daily physical activity could reduce WC and improve psychological patterns. Further research on these findings remains a priority on Ecuador’s public health agenda.

GGMs introduce a restricted selection criterion, retaining only certain variables in the final model and forcing others to zero to facilitate the interpretation of the data structure [42]. However, it is acknowledged that a potential limitation is the requirement for the data to follow a Gaussian distribution, which is not met by all variables in the health pattern, or that the researcher considers all necessary categories for a comprehensive characterization.

It is also considered important to take into account the results of other studies that contrast with the findings presented. At the University of Ghana, it was determined that the prevalence of overweight/obesity among university students was high: 33.8% among female students and 17.0% among male students. A significant association was found between overweight/obesity and potential factors, including sex, family history of obesity, and university level, showing a relationship with this study [43]. A literature review on students in India found that most students were male, with an average age of 18.57 years, and 19% of students were overweight. Given the sex-specific and WC cut-off points, around 5% and 21% of the students had a substantially higher risk of metabolic complications [44], which relates to what is described in Table 1. Additionally, it has been observed that the university environment increases the risk of weight gain [45], identifying a similarity with what is presented in Table 1.

According to Spiegelman [46], metabolic diseases have been considered a consequence of “modern civilization”, making abundant, nutrient-rich food available while reducing physical labor. However, obesity has been largely (and inadequately) attributed solely to “excessive energy intake”, which consequently generates an “energy imbalance” that leads to an excessive accumulation of fat. This identifies a relationship with daily caloric intake, where the median consumption exceeds the recommendation for the young population, which is 2000 kcal/day.

The available evidence highlights that obesity is a multifactorial condition related to diet, low physical activity levels, nutrient distribution disorders, hypothyroidism, depression, stress, bulimia, or genetic, epigenetic, ethnic, or social environment factors [47,48], causes that have also been identified in Networks D, H, and L.

However, in all cases, there is an excessive ectopic accumulation of fat, while (at least) lipid and carbohydrate metabolism is altered, and insulin function is impaired [49], showing a relationship with what is presented in Networks H and L.

One of the important findings of the study is the behavior of the blood pressure variable. Several publications show that obesity generates a higher risk of hypertension [50,51], with a similar pattern to that identified in Network C, where higher triglyceride, cholesterol, and sodium levels are associated with higher blood pressure values.

Recent evidence suggests that there is an association between psychological factors and the etiology of obesity [52], consistent with the evidence from other studies. Obesity may not only lead to physical illnesses but can also be accompanied by psychological disorders and social problems such as low self-esteem, depression, and social stigma [14].

Other studies have shown that depression and anxiety scores had a direct correlation with BMI, and scores in the overweight and obese groups were significantly higher than those in the normal-weight group [53,54]. These results supported the findings shown in Networks C and D. A similar condition was described by Milano et al., who revealed that there is a significant relationship between obesity-related depression, metabolic disturbances, and the presence of proinflammatory cytokines [15].

Multiple micronutrient deficiencies are a global problem and extend to various population groups, including seemingly healthy young people [55]. There is increasing evidence supporting the existence of an association between obesity and iron deficiency [56]. The main mechanism linking obesity and iron deficiency is low-grade systemic inflammation, observed in people with obesity [57]. This information relates to the behavior of the iron variable, presented in Networks B, D, J, and Table 2, which shows that the iron intake in the patients’ diet is lower than the recommended levels.

There are factors associated with the transition from MHO to MUO, indicating decreased insulin sensitivity and increased fasting glucose [58], correlating with what was identified in Network D. The risk of developing type 2 diabetes is much lower in those with MHO than in those with MUO [59] and is directly related to the number of metabolic abnormalities at baseline [60], also showing a relationship with what is described in Table 2. Insulin sensitivity is higher in people with MHO than in those with MUO, as manifested by higher fasting plasma insulin concentrations, blood glucose concentrations during an oral glucose tolerance test, and HOMA-IR values [61,62,63].

There is evidence from some studies [64,65] that the consumption of specific types of foods differs between the MHO and MUO groups; MHO was associated with lower intake of sugar, sugary drinks, and saturated fats, and higher intake of whole fruits, whole grains, and plant-based protein sources, showing a relationship with what is presented in Table 2 regarding the dietary patterns of the two groups.

## 5. Conclusions

Graphical models are presented as a novel tool for exploring relationships between elements and variables in large datasets, especially when researchers include variables from multiple theories that have not been studied together before.

It has been demonstrated that this method of analysis benefits researchers by providing a global view of all the relationships between the variables included in a dataset. Additionally, it reveals the strength of the relationships between variables and allows for the comparison of differences and similarities in these relationships to establish an understanding within the dataset across groups. This work surpasses the limits of previous research, as no network analysis has been reported in Ecuador among the university population with overweight and obesity.

The findings of this study revealed a difference in the network graphs identified by GGM between the MUO and MHO groups. Overall, GGM can provide further understanding of the relationships between dietary patterns, psychological factors, and metabolic health in university students.

Similarly, these models offer benefits for scientific argumentation when experts from multiple disciplines contribute to improving the understanding of public health issues or other highly complex problems.

Moreover, this method not only provides some initial insights into the relationships between elements and variables but also disrupts traditional analysis, paving the way for future research to test new datasets. Therefore, Gaussian graphical models allow for the exploration and understanding of the relationships between variables in a phenomenon as complex as obesity in a young population.

The study has identified new insights into depression, anxiety, and stress as risk factors for obesity. These findings suggest that health education programs focused on overweight and obesity also need to be integrated into mental health programs to ensure adherence and the well-being of participants.

## Figures and Tables

**Table 1:** Descriptive statistics and normality test of the data.

					Shapiro-Wilk	
Variable	Median	DE	Minimum	Maximum	W	*p*
Age	21.00	2.51	18	28	0.906	<0.001
Anthropometric						
Weight (kg)	74.80	11.14	52.5	113.50	0.967	<0.001
Height (m)	1.63	0.09	1.41	1.88	0.991	0.194
IMC	27.60	2.76	25.1	42.30	0.852	<0.001
Cardiometabolic risk factors						
Waist circumference (cm)	90.00	7.53	76	112	0.967	<0.001
Systolic blood pressure (mm Hg)	120.00	8.54	90	139	0.854	<0.001
Diastolic blood pressure (mm Hg)	80.00	6.45	60	100	0.754	<0.001
Glucose (mg/dL)	95.86	10.77	75.31	130.10	0.804	<0.001
Insulin (U/mL)	10.41	2.76	2.4	22.62	0.999	<0.001
Cholesterol (mg/dL)	195.73	55.41	93.87	456.24	0.888	<0.001
Triglycerides (mg/dL)	157.24	50.72	52	385.44	0.882	<0.001
HDL (mg/dL)	44.25	3.10	40	53.19	0.958	<0.001
HOMA	2.46	0.74	0.57	5.20	0.971	<0.001
Intake pattern						
Kcal/día	2249.94	239.80	1687	2956.57	0.961	<0.001
Cholesterol (mg)	355.99	101.63	139.62	733.81	0.945	<0.001
Fiber (g)	21.24	6.25	7.58	35.36	0.989	0.067
Proteins(g)	72.41	19.48	25	139.20	0.986	0.021
Carbohydrates (g)	297.75	49.29	165.51	448.40	0.984	0.011
Fats (g)	87.05	12.53	60.07	131.64	0.972	<0.001
Monounsaturated fatty acids (g)	40.99	12.01	9.46	78.38	0.935	<0.001
Polyunsaturated fatty acids (g)	8.80	4.12	1.01	49.69	0.655	<0.001
Saturated fatty acids (g)	29.75	9.57	12.85	54.61	0.963	<0.001
Water (mL)	1493.89	262.60	857.88	1994.96	0.983	0.006
Vitamin A (μg)	633.29	367.14	222.56	3961.87	0.645	<0.001
Vitamin D (μg)	2.15	0.73	0	529.50	0.069	<0.001
Vitamin E (mg)	9.84	0.80	2.06	53.33	0.664	<0.001
Thiamine (Vitamin B1) (mg)	1.19	0.90	0.45	6.01	0.702	<0.001
Riboflavin (Vitamin B2) (mg)	1.87	8.02	0.64	8.02	0.591	<0.001
Niacin (Vitamin B3) (mg)	18.27	94.89	5.88	39.61	0.964	<0.001
Pantothenic Acid (Vitamin B5) (mg)	3.95	34.85	1.31	9.51	0.911	<0.001
Pyridoxine (Vitamin B6) (mg)	1.58	5.98	0.71	8.41	0.604	<0.001
Biotin (Vitamin B8) (μg)	3.57	6.74	0.05	9.69	0.987	0.030
Folic Acid (Vitamin B9) (μg)	302.54	1.69	78.69	670.29	0.959	<0.001
Cobalamin (Vitamin B12) (μg)	13.08	1.67	1.73	42.05	0.900	<0.001
Vitamin C (mg)	151.04	98.94	12.45	403.44	0.944	<0.001
Sodium (mg)	7099.66	2332.11	861.56	14,663.46	0.978	0.001
Potassium (mg)	3362.47	1132.61	1285.65	8695.05	0.936	<0.001
Calcium (mg)	1234.77	359.12	550.51	3872.18	0.897	<0.001
Phosphorus (mg)	1451.23	376.28	546.80	2918.23	0.971	<0.001
Magnesium (mg)	384.68	119.86	168.47	1115.64	0.931	<0.001
Iron (mg)	15.40	6.54	4.45	103.20	0.381	<0.001
Zinc (mg)	9.46	3.09	2.62	20.17	0.984	0.009
Iodine (μg)	144.19	1093.68	32.72	16,698	0.060	<0.001
Copper (mg)	1.02	0.40	0.18	2.74	0.957	<0.001
Chlorine (mg)	1810.66	1835.37	408.88	9127.22	0.648	<0.001
Manganese (mg)	2.76	1.68	0.33	6.96	0.902	<0.001
Selenium (μg)	55.78	517.56	29.90	5666	0.079	<0.001
Psychological Pattern						
Depression score	5.00	1.27	5	11	0.758	<0.001
Stress score	11.00	2.87	8	17	0.872	<0.001
Anxiety score	5.00	1.63	4	9	0.809	<0.001

**Table 2:** Comparison of MHO and MUO groups.

Variable	MHO N = 66 (29%)	MUO N = 164 (71%)	*p*-Value	q-Value
Age (years)	20.00 (19.00, 22.00)	21.00 (19.00, 23.00)	0.25	0.40
Weight (kg)	72 (63, 78)	76 (68, 83)	0.003 *	0.012
Height (m)	1.64 (1.55, 1.68)	1.62 (1.56, 1.69)	0.91	0.93
Waist circumference (cm)	86 (82, 91)	90 (86, 96)	<0.001 *	<0.001
Systolic blood pressure (mm Hg)			<0.001 *	<0.001
	4 (6.1%)	0 (0%)		
	1 (1.5%)	0 (0%)		
	26 (39%)	60 (37%)		
	35 (53%)	50 (30%)		
	0 (0%)	7 (4.3%)		
	0 (0%)	46 (28%)		
	0 (0%)	1 (0.6%)		
Diastolic blood pressure (mm Hg)			0.004 *	0.014
	7 (11%)	1 (0.6%)		
	24 (36%)	61 (37%)		
	35 (53%)	96 (59%)		
	0 (0%)	3 (1.8%)		
	0 (0%)	3 (1.8%)		
Glucose (mg/dL)	93 (90, 96)	97 (93, 104)	<0.001 *	<0.001
Insulin (U/mL)	9.78 (8.70, 10.40)	10.64 (10.21, 11.46)	<0.001 *	<0.001
Cholesterol (mg/dL)	155 (142, 172)	208 (186, 230)	<0.001 *	<0.001
Triglycerides (mg/dL)	101 (90, 112)	162 (156, 175)	<0.001 *	<0.001
HDL (mg/dL)	47.09 (44.78, 49.16)	43.73 (40.93, 45.35)	<0.001 *	<0.001
HOMA	2.24 (1.94, 2.42)	2.53 (2.30, 3.14)	<0.001 *	<0.001
IMC	26.65 (25.90, 27.58)	28.20 (26.78, 30.47)	<0.001 *	<0.001
Kcal/día	2.258 (2.165, 2.318)	2.238 (2.104, 2.447)	0.81	0.90
Cholesterol (mg)	336 (285, 399)	369 (317, 432)	0.014	0.038
Fiber (g)	23 (20, 29)	21 (17, 25)	0.006	0.017
Proteins (g)	69 (55, 82)	74 (62, 85)	0.078	0.17
Carbohydrates (g)	298 (270, 325)	297 (265, 330)	0.83	0.90
Fats (g)	88 (85, 94)	86 (79, 96)	0.17	0.30
Monounsaturated fatty acids (g)	42 (39, 47)	40 (35, 45)	0.035	0.091
Polyunsaturated fatty acids (g)	8.86 (7.20, 9.88)	8.77 (7.49, 10.17)	0.51	0.66
Saturated fatty acids (g)	27 (22, 37)	30 (24, 39)	0.15	0.29
Water (mL)	1.551 (1.299, 1.738)	1.475 (1.267, 1.667)	0.060	0.15
Vitamin A (μg)	657 (500, 870)	612 (502, 881)	0.45	0.61
Thiamine (Vitamin B1) (mg)	1.13 (0.99, 1.42)	1.22 (1.02, 1.71)	0.073	0.16
Riboflavin (Vitamin B2) (mg)	1.88 (1.66, 2.06)	1.87 (1.65, 2.10)	0.72	0.87
Pyridoxine (VitaminaB6) (mg)	1.56 (1.36, 1.76)	1.60 (1.41, 2.02)	0.36	0.51
Cobalamin (Vitamin B12) (μg)	15 (3, 20)	13 (4, 19)	0.78	0.90
Vitamin C (mg)	152 (80, 213)	151 (94, 227)	0.26	0.40
Vitamin D (μg)	2.24 (0.71, 2.82)	2.02 (1.33, 2.67)	0.86	0.91
Vitamin E (mg)	9.7 (6.4, 13.1)	10.0 (8.0, 12.1)	0.81	0.90
Niacin (Vitamin B3) (mg)	17.8 (15.6, 22.2)	18.6 (13.8, 25.3)	0.35	0.51
Pantothenic Acid (Vitamin B5)	3.63 (3.14, 4.93)	4.00 (3.33, 5.01)	0.26	0.40
Biotin (Vitamin B8)	3.33 (2.56, 4.49)	3.77 (2.79, 4.87)	0.071	0.16
Folic Acid (Vitamin B9) (μg)	311 (244, 366)	300 (231, 347)	0.29	0.44
Sodium (mg)	6.608 (5.590, 7.638)	7.652 (5.852, 9.603)	0.011	0.032
Potassium (mg)	3.500 (2.504, 3.782)	3.316 (2.732, 4.003)	0.99	0.99
Calcium (mg)	1.281 (1.140, 1.484)	1.224 (1.093, 1.441)	0.26	0.40
Phosphorus (mg)	1.429 (1.291, 1.541)	1.465 (1.266, 1.702)	0.22	0.38
Magnesium (mg)	383 (328, 434)	385 (324, 455)	0.61	0.75
Iron (mg)	15.4 (14.0, 16.1)	15.4 (13.8, 17.1)	0.48	0.63
Zinc (mg)	8.57 (6.37, 11.26)	9.73 (7.39, 11.78)	0.081	0.17
Iodine (μg)	99 (60, 156)	155 (94, 179)	<0.001 *	<0.001
Copper (mg)	0.97 (0.69, 1.09)	1.04 (0.83, 1.22)	0.10	0.19
Chlorine (mg)	1.787 (1.511, 2.509)	1.813 (1.496, 2.467)	0.90	0.93
Manganese (mg)	1.37 (1.07, 2.90)	2.88 (1.52, 3.76)	<0.001 *	<0.001
Selenium (μg)	56 (47, 61)	56 (48, 67)	0.53	0.66
Depression score			0.001 *	0.004
	48 (73%)	71 (43%)		
	9 (14%)	25 (15%)		
	8 (12%)	45 (27%)		
	1 (1.5%)	15 (9.1%)		
	0 (0%)	4 (2.4%)		
	0 (0%)	4 (2.4%)		
Stress score	8.00 (8.00, 10.00)	11.00 (10.00, 14.00)	<0.001 *	<0.001
Anxiety score				
	48 (73%)	47 (29%)		
	7 (11%)	40 (24%)		
	0 (0%)	14 (8.5%)		
	5 (7.6%)	24 (15%)		
	5 (7.6%)	34 (21%)		
	0 (0%)	4 (2.4%)		
	0 (0%)	4 (2.4%)		
Stress score	8.00 (8.00, 10.00)	11.00 (10.00, 14.00)	<0.001 *	<0.001
Anxiety score				
	48 (73%)	47 (29%)		
	7 (11%)	40 (24%)		
	0 (0%)	14 (8.5%)		
	5 (7.6%)	24 (15%)		
	5 (7.6%)	34 (21%)		
	1 (1.5%)	5 (3.0%)		

* *p* < 0.005 statistically significant difference.

## Data Availability

Access to the study information will be provided by the authors, through the e-mails declared and sent to the researchers, in compliance with the Ethics Committee’s provision on the anonymization of information.

## Supplementary Materials

The following supporting information can be downloaded at: https://www.mdpi.com/article/10.3390/nu16172924/s1, Table S1. Relationship of variables Overweight and Global Obesity; Table S2. List of MUO population variables; Table S3. List of MHO population variables.

## Appendix A

**Table A1:** Variable Coding.

Variable	Code
Age	C1
Sex at Birth	C2
Marital Status	C3
Academic Unit	C4
Mayor	C5
Study Schedule	C6
Socioeconomic Status	C7
Depression Level Categorization	C8
Anxiety Level Categorization	C9
Stress Level Categorization	C10
Physical Activity Level	C11
Weight	C12
Height	C13
Nutritional Status	C14
Waist Circumference	C15
Systolic Blood Pressure	C16
Diastolic Blood Pressure	C17
Glucose	C18
Insulin	C19
Cholesterol	C20
Triglycerides	C21
HDL	C22
HOMA	C23
MUO and MHO Categorization	C24
BMI (Body Mass Index)	C25
Blood Pressure Categorization	C26
Waist Circumference Categorization	C27
MUO and MHO Categorization 2 by HOMA	C28
Kcal/day	C29
Cholesterol	C30
Fiber	C31
Proteins	C32
Carbohydrates	C33
Fats	C34
Monounsaturated Fatty Acids	C35
Polyunsaturated Fatty Acids	C36
Saturated Fatty Acids	C37
Water	C38
Vitamin A	C39
Vitamin D	C45
Vitamin E	C46
Thiamine (Vitamin B1)	C40
Riboflavin (Vitamin B2)	C41
Niacin (Vitamin B3)	C47
Pantothenic Acid (Vitamin B5)	C48
Pyridoxine (Vitamin B6)	C42
Biotin (Vitamin B8)	C49
Folic Acid (Vitamin B9)	C50
Cobalamin (Vitamin B12)	C43
Vitamin C	C44
Sodium	C51
Potassium	C52
Calcium	C53
Phosphorus	C54
Magnesium	C55
Iron	C56
Zinc	C57
Iodine	C58
Copper	C59
Chlorine	C60
Manganese	C61
Selenium	C62
Depression score	C63
Stress score	C64
Anxiety score	C65
