# Supplementary material for: Irruption of Network Analysis to Explain Dietary, Psychological and Nutritional Patterns and Metabolic Health Status in Metabolically Healthy and Unhealthy Overweight and Obese University Students: Ecuadorian Case
Source: Nutrients. 2024 Sep 1;16(17):2924. doi: 10.3390/nu16172924 (PMC11397439; doi:10.3390/nu16172924)
Supplement: Supplementary file 1 [file nutrients-16-02924-s001.zip › nutrients-3146474-supplementary.pdf]

Table S1. Relationship of variables Overweight and Global Obesity

Code	Code	Rho de Sperman	p
C 63	C 64	0.548	< 0.001
C 59	C 60	0.575	< 0.001
C 56	C 59	0.424	< 0.001
C 55	C 56	0.488	< 0.001
C 55	C 59	0.535	< 0.001
C 54	C 55	0.475	< 0.001
C 54	C 56	0.473	< 0.001
C 52	C 59	0.422	< 0.001
C 50	C 59	0.433	< 0.001
C 49	C 52	0.52	< 0.001
C 49	C 54	0.419	< 0.001
C 48	C 50	0.576	< 0.001
C 48	C 52	0.54	< 0.001
C 48	C 54	0.416	< 0.001
C 47	C 56	0.414	< 0.001
C 47	C 62	0.562	< 0.001
C 44	C 46	0.454	< 0.001
C 44	C 48	0.457	< 0.001
C 44	C 50	0.585	< 0.001
C 44	C 54	0.444	< 0.001
C 44	C 55	0.469	< 0.001
C 43	C 45	0.4	< 0.001
C 42	C 46	0.418	< 0.001
C 42	C 47	0.544	< 0.001
C 42	C 48	0.522	< 0.001
C 42	C 52	0.431	< 0.001
C 42	C 54	0.532	< 0.001
C 42	C 55	0.476	< 0.001
C 42	C 56	0.467	< 0.001
C 42	C 59	0.458	< 0.001
C 41	C 42	0.47	< 0.001
C 41	C 45	0.453	< 0.001
C 41	C 54	0.531	< 0.001
C 40	C 44	0.483	< 0.001
C 40	C 47	0.44	< 0.001
C 40	C 48	0.519	< 0.001
C 40	C 52	0.444	< 0.001
C 40	C 55	0.41	< 0.001
C 40	C 56	0.426	< 0.001
C 40	C 59	0.53	< 0.001
C 39	C 41	0.426	< 0.001
C 39	C 42	0.405	< 0.001
C 39	C 46	0.436	< 0.001
C 39	C 53	0.524	< 0.001
C 37	C 40	0.44	< 0.001
C 37	C 54	0.454	< 0.001
C 37	C 55	0.415	< 0.001
C 36	C 46	0.553	< 0.001
C 35	C 39	0.481	< 0.001

C 35	C 46	0.511	< 0.001
C 35	C 53	0.463	< 0.001
C 33	C 37	0.554	< 0.001
C 33	C 40	0.433	< 0.001
C 33	C 54	0.431	< 0.001
C 31	C 50	0.423	< 0.001
C 30	C 48	0.471	< 0.001
C 30	C 54	0.492	< 0.001
C 30	C 55	0.438	< 0.001
C 30	C 59	0.403	< 0.001
C 29	C 36	0.401	< 0.001
C 29	C 37	0.517	< 0.001
C 22	C 23	-0.404	< 0.001
C 21	C 22	-0.519	< 0.001
C 21	C 23	0.447	< 0.001
C 20	C 22	-0.468	< 0.001
C 20	C 23	0.43	< 0.001
C 18	C 19	0.414	< 0.001
C 18	C 20	0.502	< 0.001
C 18	C 21	0.402	< 0.001
C 18	C 25	0.479	< 0.001
C 15	C 22	-0.449	< 0.001
C 15	C 25	0.554	< 0.001
C 13	C 15	0.531	< 0.001
C 12	C 16	0.405	< 0.001
C 12	C 25	0.559	< 0.001
C 63	C 65	0.618	< 0.001
C 52	C 54	0.6	< 0.001
C 52	C 55	0.737	< 0.001
C 50	C 52	0.64	< 0.001
C 50	C 55	0.65	< 0.001
C 48	C 55	0.624	< 0.001
C 48	C 59	0.614	< 0.001
C 44	C 49	0.631	< 0.001
C 44	C 52	0.687	< 0.001
C 41	C 53	0.605	< 0.001
C 40	C 42	0.633	< 0.001
C 40	C 54	0.64	< 0.001
C 29	C 33	0.701	< 0.001
C 29	C 34	0.605	< 0.001
C 20	C 21	0.728	< 0.001
C 18	C 23	0.628	< 0.001
C 12	C 13	0.785	< 0.001
C 12	C 15	0.765	< 0.001

Table S2. List of MUO population variables

**Code**	**Code**	**Rho de Sperman**	**p**
C1	C22	-0.299	< 0.001
C1	C40	0.234	0.003
C1	C42	0.228	0.003
C1	C48	0.223	0.004

C1	C52	0.232	0.003
C1	C55	0.228	0.003
C1	C56	0.250	0.001
C1	C64	0.246	0.002
C12	C13	0.762	< 0.001
C12	C15	0.807	< 0.001
C12	C16	0.390	< 0.001
C12	C17	0.278	< 0.001
C12	C18	0.382	< 0.001
C12	C20	0.288	< 0.001
C12	C21	0.256	< 0.001
C12	C22	-0.350	< 0.001
C12	C23	0.306	< 0.001
C12	C25	0.575	< 0.001
C13	C15	0.563	< 0.001
C13	C16	0.229	0.003
C13	C22	-0.266	< 0.001
C15	C16	0.364	< 0.001
C15	C17	0.268	< 0.001
C15	C18	0.397	< 0.001
C15	C20	0.327	< 0.001
C15	C21	0.287	< 0.001
C15	C22	-0.426	< 0.001
C15	C23	0.308	< 0.001
C15	C25	0.547	< 0.001
C16	C17	0.808	< 0.001
C16	C18	0.414	< 0.001
C16	C19	0.241	0.002
C16	C20	0.246	0.001
C16	C21	0.235	0.002
C16	C23	0.356	0.001
C16	C25	0.321	< 0.001
C17	C18	0.339	< 0.001
C17	C19	0.245	0.002
C17	C23	0.323	< 0.001
C17	C25	0.269	< 0.001
C17	C58	-0.236	0.002
C18	C19	0.383	< 0.001
C18	C20	0.439	< 0.001
C18	C21	0.242	0.002
C18	C22	-0.258	< 0.001
C18	C23	0.656	< 0.001
C18	C25	0.531	< 0.001
C18	C63	0.299	< 0.001
C18	C64	0.229	0.003
C19	C20	0.222	0.004
C19	C21	0.363	< 0.001
C19	C22	-0.259	< 0.001
C19	C23	0.893	< 0.001
C19	C25	0.321	< 0.001
C20	C21	0.581	< 0.001

C20	C22	-0.308	< 0.001
C20	C23	0.366	< 0.001
C20	C25	0.298	< 0.001
C21	C22	-0.346	< 0.001
C21	C23	0.377	< 0.001
C22	C23	-0.277	< 0.001
C22	C38	-0.274	< 0.001
C22	C 52	-0.221	0.004
C23	C25	0.427	< 0.001
C23	C54	-0.217	0.005
C25	C60	-0.238	0.002
C25	C63	0.235	0.002
C25	C64	0.239	0.002
C25	C65	0.227	0.004
C29	C31	-0.223	0.004
C29	C32	0.267	< 0.001
C29	C33	0.721	< 0.001
C29	C34	0.636	< 0.001
C29	C35	0.269	< 0.001
C29	C36	0.437	< 0.001
C29	C37	0.577	< 0.001
C29	C40	0.491	< 0.001
C29	C41	0.255	< 0.001
C29	C42	0.253	< 0.001
C29	C46	0.383	< 0.001
C29	C47	0.257	< 0.001
C29	C54	0.416	< 0.001
C29	C56	0.379	< 0.001
C29	C58	0.298	< 0.001
C29	C59	0.290	< 0.001
C29	C61	0.362	< 0.001
C29	C62	0.316	< 0.001
C30	C37	0.251	< 0.001
C30	C40	0.373	< 0.001
C30	C41	0.267	< 0.001
C30	C42	0.332	< 0.001
C30	C44	0.256	< 0.001
C30	C48	0.522	< 0.001
C30	C49	0.304	< 0.001
C30	C50	0.236	< 0.001
C30	C52	0.414	< 0.001
C30	C54	0.546	< 0.001
C30	C55	0.478	< 0.001
C30	C56	0.394	< 0.001
C30	C57	0.295	< 0.001
C30	C58	0.280	< 0.001
C30	C59	0.401	< 0.001
C31	C50	0.407	< 0.001
C31	C61	-0.417	< 0.001
C32	C34	0.405	< 0.001
C33	C37	0.584	< 0.001

C33	C40	0.487	< 0.001
C35	C39	0.469	< 0.001
C35	C46	0.486	< 0.001
C35	C53	0.444	< 0.001
C36	C46	0.588	< 0.001
C37	C40	0.521	< 0.001
C39	C41	0.468	< 0.001
C39	C42	0.437	< 0.001
C39	C44	0.426	< 0.001
C39	C46	0.409	< 0.001
C39	C53	0.559	< 0.001
C40	C42	0.681	< 0.001
C40	C44	0.403	< 0.001
C40	C46	0.425	< 0.001
C40	C47	0.538	< 0.001
C40	C48	0.500	< 0.001
C40	C52	0.464	< 0.001
C40	C54	0.676	< 0.001
C40	C55	0.433	< 0.001
C40	C56	0.470	< 0.001
C40	C59	0.565	< 0.001
C40	C62	0.421	< 0.001
C41	C42	0.464	< 0.001
C41	C45	0.471	< 0.001
C41	C53	0.675	< 0.001
C41	C54	0.580	< 0.001
C42	C46	0.470	< 0.001
C42	C47	0.547	< 0.001
C42	C48	0.482	< 0.001
C42	C52	0.428	< 0.001
C42	C54	0.521	< 0.001
C42	C55	0.477	< 0.001
C42	C56	0.459	< 0.001
C42	C59	0.521	< 0.001
C44	C48	0.470	< 0.001
C44	C49	0.657	< 0.001
C44	C50	0.617	< 0.001
C44	C52	0.686	< 0.001
C44	C54	0.458	< 0.001
C44	C55	0.488	< 0.001
C47	C56	0.430	< 0.001
C47	C62	0.598	< 0.001
C48	C50	0.555	< 0.001
C48	C52	0.530	< 0.001
C48	C54	0.451	< 0.001
C48	C55	0.628	< 0.001
C48	C59	0.576	< 0.001
C49	C52	0.569	< 0.001
C49	C54	0.477	< 0.001
C49	C55	0.414	< 0.001
C50	C52	0.712	< 0.001

C50	C55	0.699	< 0.001
C50	C59	0.419	< 0.001
C52	C54	0.621	< 0.001
C52	C55	0.759	< 0.001
C52	C59	0.456	< 0.001
C54	C55	0.506	< 0.001
C54	C56	0.499	< 0.001
C55	C57	0.478	< 0.001
C55	C60	0.566	< 0.001
C56	C59	0.447	< 0.001
C59	C60	0.530	< 0.001
C63	C64	0.515	< 0.001
C63	C65	0.574	< 0.001

Table S3. List of MHO population variables.

**Code**	**Code**	**Rho de Sperman**	**p**
C12	C25	0.466	< 0.001
C12	C15	0.619	< 0.001
C12	C13	0.888	< 0.001
C13	C15	0.532	< 0.001
C16	C17	0.988	< 0.001
C18	C23	0.423	< 0.001
C19	C23	0.945	< 0.001
C25	C44	0.4	< 0.001
C25	C52	0.407	< 0.001
C29	C33	0.625	< 0.001
C29	C34	0.531	< 0.001
C29	C54	0.468	< 0.001
C30	C57	0.456	< 0.001
C31	C35	0.452	< 0.001
C31	C37	0.408	< 0.001
C31	C44	0.448	< 0.001
C31	C50	0.425	< 0.001
C31	C51	-0.444	< 0.001
C31	C53	0.504	< 0.001
C31	C55	0.447	< 0.001
C32	C33	-0.516	< 0.001
C32	C59	0.41	< 0.001
C32	C60	0.428	< 0.001
C33	C37	0.472	< 0.001
C33	C54	0.544	< 0.001
C35	C36	0.535	< 0.001
C35	C38	0.541	< 0.001
C35	C39	0.545	< 0.001
C35	C46	0.605	< 0.001
C35	C53	0.508	< 0.001
C36	C43	0.509	< 0.001
C36	C45	0.548	< 0.001
C36	C46	0.485	< 0.001
C37	C53	0.542	< 0.001
C37	C55	0.562	< 0.001

C38	C39	0.513	< 0.001
C38	C43	0.498	< 0.001
C38	C45	0.406	< 0.001
C38	C54	0.482	< 0.001
C38	C56	0.57	< 0.001
C39	C45	0.501	< 0.001
C39	C53	0.429	< 0.001
C40	C42	0.436	< 0.001
C40	C44	0.624	< 0.001
C40	C48	0.526	< 0.001
C40	C50	0.478	< 0.001
C40	C54	0.459	< 0.001
C41	C42	0.517	< 0.001
C41	C46	0.434	< 0.001
C41	C54	0.402	< 0.001
C41	C58	0.458	< 0.001
C42	C47	0.49	< 0.001
C42	C48	0.6	< 0.001
C42	C52	0.465	< 0.001
C42	C54	0.549	< 0.001
C42	C55	0.487	< 0.001
C42	C56	0.46	< 0.001
C43	C45	0.558	< 0.001
C44	C46	0.599	< 0.001
C44	C48	0.442	< 0.001
C44	C49	0.578	< 0.001
C44	C50	0.549	< 0.001
C44	C52	0.645	< 0.001
C44	C55	0.41	< 0.001
C45	C50	-0.403	< 0.001
C46	C49	0.467	< 0.001
C46	C52	0.446	< 0.001
C47	C62	0.437	< 0.001
C48	C50	0.652	< 0.001
C48	C52	0.626	< 0.001
C48	C55	0.617	< 0.001
C48	C56	0.404	< 0.001
C48	C57	0.408	< 0.001
C48	C59	0.686	< 0.001
C50	C52	0.465	< 0.001
C50	C55	0.533	< 0.001
C50	C56	0.432	< 0.001
C50	C59	0.501	< 0.001
C52	C54	0.588	< 0.001
C52	C55	0.706	< 0.001
C52	C58	0.419	< 0.001
C53	C55	0.479	< 0.001
C55	C56	0.53	< 0.001
C55	C57	0.419	< 0.001
C55	C59	0.457	< 0.001
C57	C58	0.481	< 0.001

C59	C60	0.714	< 0.001
C63	C65	0.526	< 0.001
C64	C65	0.803	< 0.001
